# Potential of Antithrombin III as a Biomarker of Antidepressive Effect in Major Depressive Disorder

**DOI:** 10.3389/fpsyt.2021.678384

**Published:** 2021-10-28

**Authors:** Ruize Song, Yachen Shi, Xianrui Li, Jianli Zhu, Hongxing Zhang, Kun Li, Bi Wang, Haisan Zhang, Yongfeng Yang, Lijuan Gao, Yang Zhao, Zhijun Zhang

**Affiliations:** ^1^Department of Neurology, School of Medicine, Affiliated ZhongDa Hospital, Southeast University, Nanjing, China; ^2^Department of Psychology, Xinxiang Medical University, Xinxiang, China; ^3^Second Affiliated Hospital of Xinxiang Medical University, Xinxiang, China; ^4^Department of Biostatistics, School of Public Health, Nanjing Medical University, Nanjing, China

**Keywords:** major depressive disorder, antithrombin III, occipital repetitive transcranial magnetic stimulation, antidepressive effect, biomarker

## Abstract

**Background:** The evaluation of treatment response to antidepressant therapy commonly depends on neuropsychologic assessments, as there are currently no suitable biomarkers. Previous research has identified a panel of increased proteins in patients with major depressive disorder (MDD), including antithrombin III (ATIII), as potential biomarkers of depression.

**Methods:** A total of 90 MDD patients were recruited. Of these, 74 patients received occipital repetitive transcranial magnetic stimulation (rTMS) as individualized, standard, or sham treatment for 5 days, and underwent the complete procedure, including clinical assessments, blood collection, and protein measurement.

**Results:** After treatment, ATIII was significantly decreased in both the individualized and standard groups (both *p* < 0.001) relative to the sham group. In the individualized group, reduction in ATIII was associated with improvements in several neuropsychological assessments. Furthermore, ATIII at baseline in the standard group and after individualized rTMS showed good performance for evaluating or predicting the response to five-day treatment (AUC = 0.771, 95% CI, 0.571–0.971; AUC = 0.875, 95% CI, 0.714–1.000, respectively) and remission at follow-up (AUC = 0.736, 95% CI, 0.529–0.943; AUC = 0.828, 95% CI, 0.656–1.000, respectively). Lastly, both baseline ATIII and change in ATIII showed good predictive value for the 24-item Hamilton Depression Rating Scale at follow-up (*p* = 0.024 and 0.023, respectively).

**Conclusion:** Our study revealed a reduction in ATIII after occipital rTMS in MDD patients and a relationship between change in ATIII and therapeutic response. Taken together, these findings provide evidence for the potential of ATIII as a biomarker for the evaluation and prediction of antidepressive effects.

## Introduction

Major depressive disorder (MDD) is a mental illness characterized by low mood and anhedonia that affects 322 million people worldwide, leading to excess disability and mortality (1–3). For the period of 2007 to 2017, MDD was the third leading cause of global years lived with disability (YLDs) (1). According to the World Health Organization, this common mental disorder will be responsible for the greatest burden of disease worldwide by 2030 (4). Both biological factors and environmental milieu are related to the occurrence of depression (5, 6); however, little is known about the physiopathological mechanisms underlying MDD. Several hypotheses have been proposed (3, 7). In one of the mainstream theories, neuroinflammation is proposed to influence MDD through abnormalities in immune cells, like microglia, astrocytes and oligodendroglia, and increases in inflammatory factors (8–12). Accumulating evidence suggests that neuroinflammation might be a mediator of MDD in connection with other possible mechanisms, such as stress, neuroendocrine system dysfunction, neurotransmitters depletion, neurogenesis defects, and intestinal flora disorder (13–17). As reported in previous studies, various anti-inflammatory treatments can improve depression symptoms, supporting that inflammatory molecules or pathways may be potential targets for novel antidepressant therapy (18, 19).

At present, MDD and antidepressive therapy response are assessed by clinical features, rather than objective indicators, duo to the lack of confirmed clinical biomarkers (3, 20, 21). As a result, it is imperative to identify and evaluate potential biomarkers of MDD. A variety of techniques have been utilized in the aim to identify such biomarkers, including genomics, transcriptomics, proteomics, metabonomics, neuroimaging, electroencephalography, etc. (22–27). In comparison to other biological parameters, proteins play direct roles in the occurrence and development of MDD and the result of antidepressive treatment (24), and thus have great application as candidate biomarkers.

In our previous proteomic study, four proteins were noted: C-reactive protein (CRP), antithrombin III (ATIII), inter-alpha-trypsin inhibitor heavy chain H4 (ITIH4), and vitamin D binding protein (VDB) (28). As an acute-phase protein, CRP is synthesized by hepatocytes and increases rapidly in response to systemic inflammation with activated innate immunity (29, 30). A multitude of studies have demonstrated that high levels of CRP are associated with MDD (31). Notably, CRP values have been used to predict the outcome of depression and resistance to standard antidepressants (32–34). ATIII is a glycoprotein mainly produced in the liver that exerts anticoagulant and anti-inflammatory effects by targeting activated thrombin and other blood coagulation factors (35, 36). In addition, ATIII can promote prostacyclin release and deactivate leukocytes to inhibit inflammation independent from coagulation (36). As reported by Stelzhammer et al., elevated levels of ATIII were detected in MDD patients after the first session of electroconvulsive therapy (ECT) (37). ITIH4 is an acute-phase protein related to inflammatory response involved in cell proliferation, migration, anti-apoptosis, and matrix stabilizing (38, 39). A genome-wide analysis reported that single nucleotide polymorphisms (SNPs) in the *ITIH4* gene were associated with bipolar disorder (40). Furthermore, according to Finseth et al., there are associations between suicide attempt and *ITIH4* (41). The main biological function of VDB, as its name suggests, is to bind and transport the specific sterol and its metabolites. Additional physiological processes regulated by this glycoprotein include actin scavenging, macrophage and osteoclast activation, and phagocytic cells chemotaxis (42–44). In prior studies, increased VDB was observed in patients with major mood disorders, comprised of both MDD and bipolar disorder (45–47). These four proteins were observed to be elevated in the depressed sample compared with controls in our previous study (28), supporting their potential to differentiate MDD. However, whether they are associated with antidepressive treatment and curative effects is unknown.

Occipital repetitive transcranial magnetic stimulation (rTMS) therapy is a novel non-invasive intervention for MDD with established safety and efficacy (48). The occipital cortex has been demonstrated to be associated with the pathophysiologic changes of MDD, which was supported by altered visual evoked potential, diminished perception of ambient light, impaired synaptic plasticity and low density of calbindin-immunoreactive GABAergic neurons in the occipital cortex in MDD patients (49–51). Moreover, a previous study suggests that selectively neural response to emotional stimuli in the occipital cortex might be a useful biomarker in identifying responders to scopolamine (52), which partly supports the role of the occipital cortex in antidepressive treatment.

This present study aimed at exploring changes in four proteins included CRP, ATIII, ITIH4, and VDB after occipital rTMS, and to assess their potential as biomarkers of the outcomes and effects of antidepressive therapy.

## Materials and Methods

### Subjects

The Ethics Committee of the Second Affiliated Hospital of Xinxiang Medical University approved this study, which was carried out at Henan Provincial Mental Hospital. All work involving human subjects followed the latest version of the Declaration of Helsinki. After the benefits and risks of the study were fully explained, a total of 90 drug naïve or drug free depressed individuals were recruited. Written informed consent was obtained from the participants or their legal guardians.

The inclusion criteria for MDD patients were as follows: (a) meeting the diagnostic criteria of MDD according to the Diagnostic and Statistical Manual of Mental Disorders, Fourth Edition (DSM-IV), assessed by two well-trained psychiatrists utilizing the Structured Clinical Interview for DSM-IV; (b) a score of >17 on the 24-item Hamilton Depression Rating Scale (HAMD-24); (c) aged 18 to 55 and drug naïve or drug free for at least 2 weeks; and (d) able to cooperate with the trial and understand the items properly.

Patients suffering from the following disorders or diseases were excluded: (a) other DSM-IV axis I or axis II disorders; (b) organic brain diseases; (c) psychotic depression; (d) endocrine diseases with abnormal biochemical indexes or organ functions; (e) treatment-resistant depression treated by electroconvulsive shock or rTMS with adequate dosage and duration; and f. diseases that result in any contraindications to magnetic resonance imaging (MRI) or rTMS. In addition, given potential effects due to alterations in hormones, female patients who were pregnant or in the puerperium or climacteric periods were excluded from this study.

### Neuropsychological Assessments

The scales for neuropsychological assessments in three dimensions (affective assessments, social-psychology assessments, and cognitive function assessments) were described in detail in our previous study (53). Briefly, to estimate the affective states of the MDD patients, the HAMD-24, Hamilton Anxiety Scale (HAMA), Beck Scale for Suicide Ideation-Chinese version-Current (BSI-CV-C), Self-Rating Depression Scale (SDS), Self-Rating Anxiety Scale (SAS), and Beck Hopelessness Scale (BHS) were conducted under unified instructions at baseline and day 1, 3, and 5 of treatment. In addition, during the 4 week follow-up study, the HAMD-24 was used once per week to evaluate the outcomes. To assess social psychology traits, patients were assessed once using the Life Event Scale (LES), Childhood Trauma Questionnaire-Short Form (CTQ-SF), family Adaptation, Partnership, Growth, Affection, Resolve (APGAR), and Self-Consciousness Scale Revised (SCSR). Cognitive functions were estimated in terms of information processing speed, executive function, and visuospatial memory and learning function (detailed evaluation tools are listed in Supplementary Table 2), and were tested at baseline and at the end of treatment.

### Occipital rTMS Treatment Regimens

The strategy and parameters of rTMS treatment were reported in the previous study (48). Briefly, MDD patients were randomly assigned to the individualized group, standard group, or sham group to receive the corresponding therapy for 5 consecutive days. For the individualized group, the coordinates of the stimulation points were personalized based on affective visual tasks completed during functional MRI. For the standard group, fixed stimulation sites were set in the left cortical visual area V1. Moreover, the following parameters were applied in rTMS treatment in this study: stimulation intensity = 90% resting motor threshold, frequency = 10 Hz, train duration = 4 s, inter-train interval = 26 s, pulse number = 1,600 in each session, total duration = 20 min, number of time = 2 times per day, total sessions = 10.

### Blood Samples

Once participants were enrolled, 5 mL of fasting venous blood was collected in a plasma separator tube (Xinle, China) between 6:00 and 10:00 in the morning. An additional 5 mL whole blood sample was drawn after the 5 day treatment. Two runs of centrifugation were completed within 2 h of blood collection. The first processing was for plasma separation with a rotational speed of 2,000 × g for 10 min at 4°C. Afterwards, the supernatant was centrifuged for the second time for purification with a rotational speed of 12,000×g for 10 min under the same conditions. Plasma samples were stored at −80°C until quantitative determination of proteins.

### Protein Quantification

Protein quantification was carried out using the enzyme-linked immunosorbent assay (ELISA) technique using commercial kits (R&D Systems, USA) according to the manufacturer's instructions. The concentration of diluted proteins was computed based on the standard curves, which was subsequently multiplied by the dilution factors to signify the actual protein level. Each sample was run in triplicate. Both the inter- and intra-assay coefficients of variation were <5%.

### Statistical Analysis

All data were analyzed using SPSS 20.0 (IBM, USA), GraphPad Prism 7.0 (GraphPad Software, USA), or SAS 9.4 (SAS Institute Inc., USA). Kolmogorov-Smirnov tests were used to assess the distributions of numerical variables. For variables with a non-normal distribution, Kruskal-Wallis tests were used to calculate the statistical significance of differences among groups. For variables with a Gaussian distribution, data were tested by one-way analyses of variance (ANOVA) with Bonferroni's *post hoc* tests. The protein levels and clinical assessments at the two time points were compared using paired *t*-tests or Wilcoxon signed-rank tests as required. In addition, Chi-squared tests were utilized to analyze categorical data. Partial correlation analyses and multivariate linear regressions were computed to determine the correlations and interactive effects, respectively. Receiver operating characteristic (ROC) curves were used to estimate and predict the antidepressive efficacy. Furthermore, a linear mixed model was established to explore predictive factors of therapeutic effects. Quantitative variables were expressed as mean [standard deviation (SD)] or median [interquartile range (IQR)], and categorical variables were expressed as absolute numbers. *P* < 0.05 was set as the level of statistical significance (two-tailed tests).

## Results

### Demographic and Clinical Characteristics

A total of 16 patients were excluded from analysis for various reasons, which are detailed in Supplementary Figure 1. Of the 74 remaining patients, 23 were assigned to the sham group, 24 were assigned to the individualized rTMS treatment group, and 27 were assigned to the standard rTMS treatment group. Of all demographic and clinical characteristics considered, the three groups only differed significantly in their drinking histories (*p* = 0.044, Supplementary Table 1).

### Neuropsychological Assessments at Baseline and End of Treatment

As shown in Supplementary Table 2, the only significant differences in neuropsychological assessment scores for the three groups at baseline were for the CTQ-SF and Stroop A (CTQ-SF: *p* = 0.010; Stroop A: *p* = 0.046). Pairwise comparisons showed that CTQ-SF scores in the standard group were significantly lower than those in the other groups (*p* = 0.027, standard group vs. individualized group; *p* = 0.028, standard group vs. sham group), while the differences in information processing speed evaluated by the Stroop A test mainly came from the contrast between the individualized group and the sham group (*p* = 0.040). Comparisons of affective scale and cognitive test scores from baseline to the end of therapy showed notable improvements in the individualized group (all *p* < 0.05). Similarly, patients treated by standard rTMS showed significant amelioration of symptoms, as shown by changes from baseline to after treatment for most assessments, except the Neuropsychological Assessment Battery: Mazes (NAB Mazes) and Brief Visuospatial Memory Test-Revised (BVMT-R) (Supplementary Table 2).

### Protein Levels at Baseline and End of Treatment

**Figure 1:**
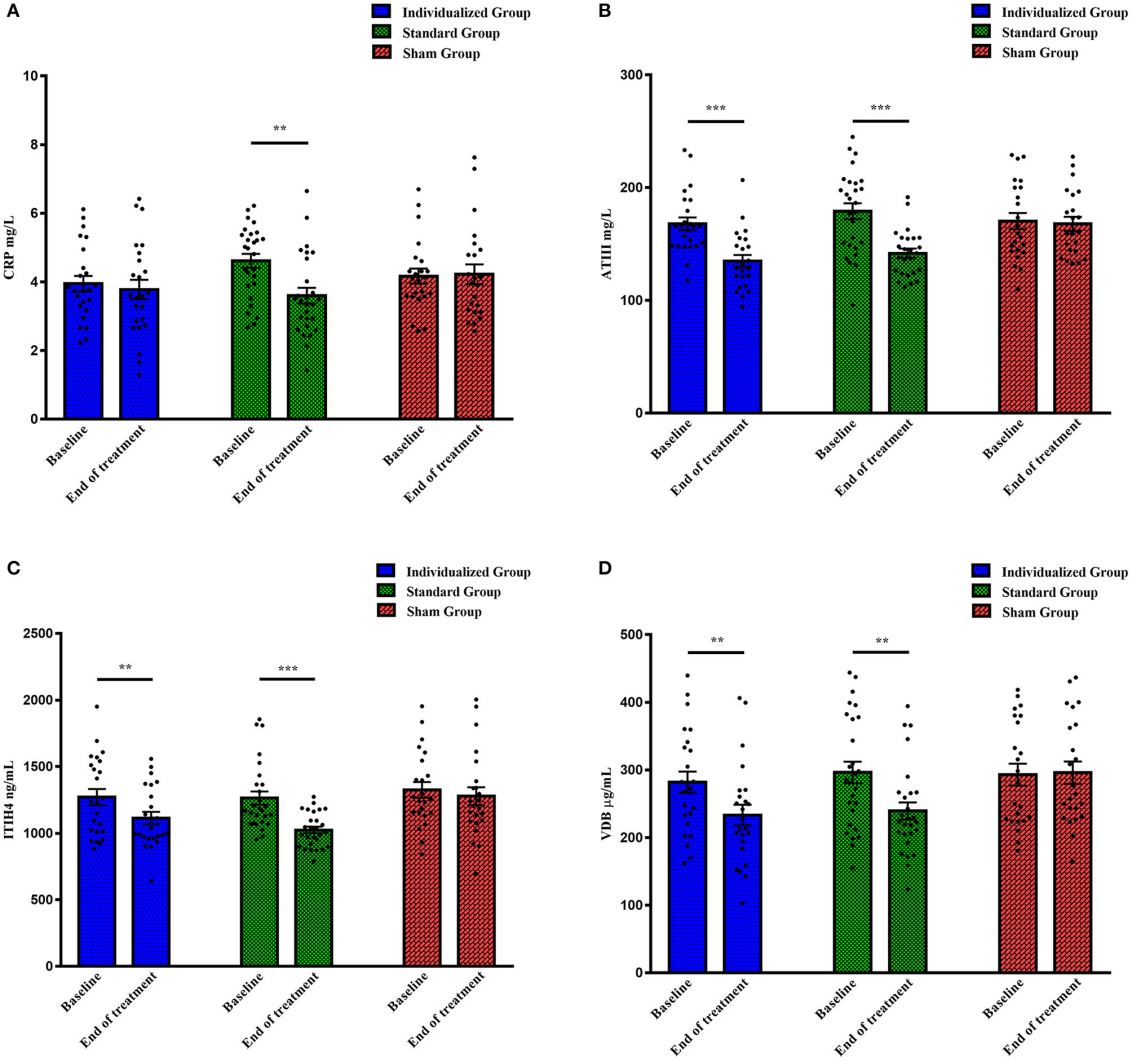
The levels of four proteins in different treatment groups at baseline and the end of treatment. **(A)** CRP levels (mg/L); **(B)** ATIII levels (mg/L); **(C)** ITIH4 levels (ng/mL); **(D)** VDB levels (μg/mL). CRP, C-reactive protein; ATIII, antithrombin III; ITIH4, inter-alpha-trypsin inhibitor heavy chain H4; VDB, vitamin D binding protein. **p < 0.01, ***p < 0.001.

Protein levels at baseline were comparable among the three groups, as shown in Figure 1 (CRP: F = 2.576, *p* = 0.083; ATIII: F = 0.802, *p* = 0.452; ITIH4: F = 0.341, *p* = 0.712; VDB: F = 0.219, *p* = 0.804). After individualized rTMS therapy, the levels of ATIII, ITIH4, and VDB significantly declined (t = 5.586, *p* < 0.001; t = 2.893, *p* = 0.008; t = 2.955, *p* = 0.007, respectively), while a downward trend with no statistical significance was observed for CRP (t = 0.503, *p* = 0.620) (Figure 1). Similarly, reductions in the expression of all proteins were demonstrated in the standard group when the whole treatment course was completed (CRP: t = 3.298, *p* = 0.003; ATIII: t = 4.523, *p* < 0.001; ITIH4: t = 4.589, *p* < 0.001; VDB: t = 3.029, *p* = 0.005) (Figure 1). In contrast to the rTMS treatment groups, as shown in Figure 1, the levels of proteins after five-day sham treatment were approximately equal to those at baseline (CRP: t = −0.148, *p* = 0.884; ATIII: t = −0.040, *p* = 0.968; ITIH4: t = 0.971, *p* = 0.342; VDB: t = −0.209, *p* = 0.836).

### Correlations of Changes in Protein Levels and Changes in Neuropsychological Assessments

**Figure 2:**
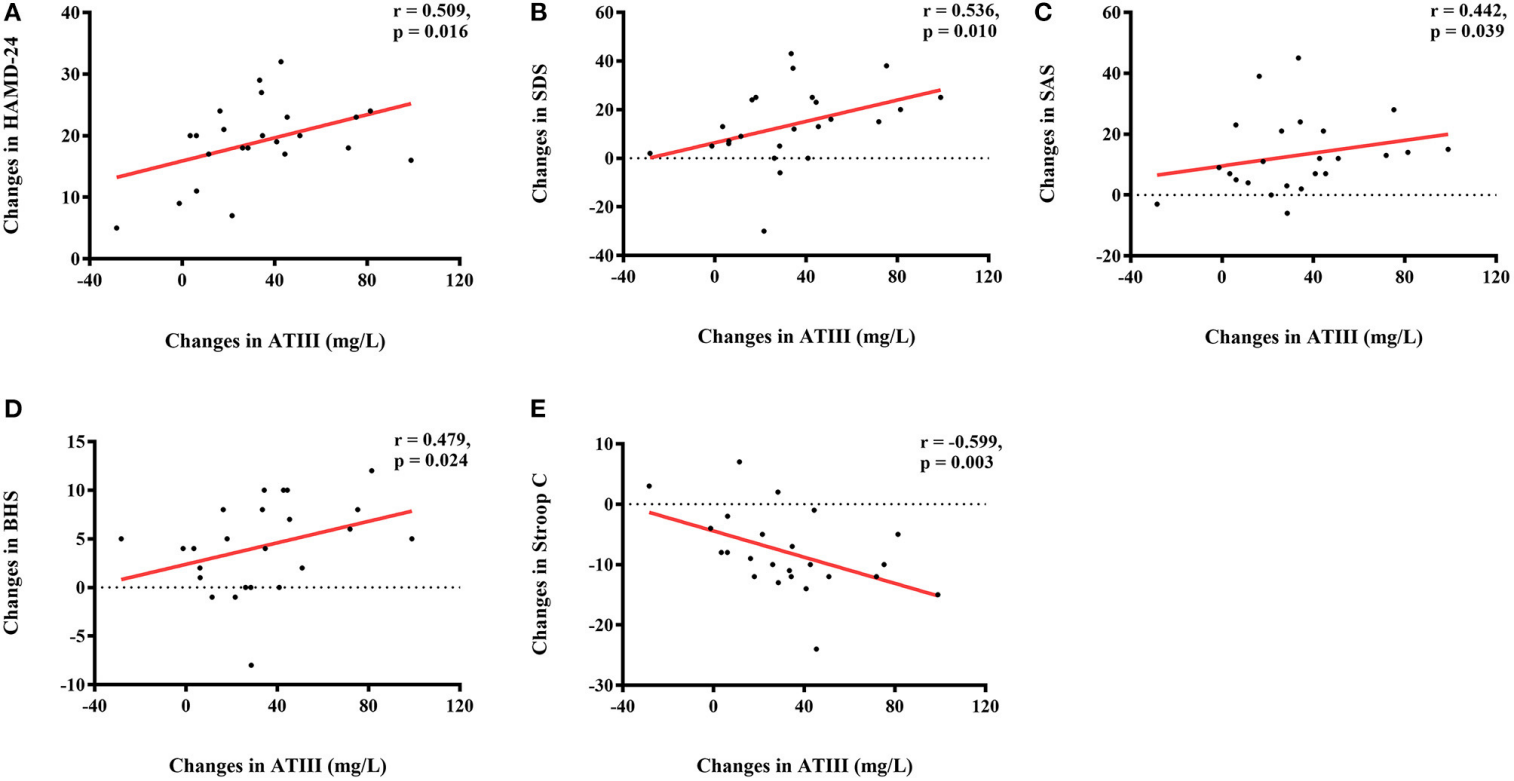
Correlations of altered ATIII (mg/L) and changes in neuropsychological assessments in the individualized group. **(A)** Positive correlation between change in ATIII level (mg/L) and change in HAMD-24; **(B)** Positive correlation between change in ATIII level (mg/L) and change in SDS; **(C)** Positive correlation between change in ATIII level (mg/L) and change in SAS; **(D)** Positive correlation between change in ATIII level (mg/L) and change in BHS; **(E)** Negative correlation between change in ATIII level (mg/L) and change in Stroop C. ATIII, antithrombin III; HAMD-24, 24-item Hamilton Depression Rating Scale; SDS, Self-Rating Depression Scale; SAS, Self-Rating Anxiety Scale; BHS, Beck Hopelessness Scale.

There were significant correlations between changes in ATIII level, but not levels of the other three proteins, and antidepressive efficacy, as evaluated by changes in affective and cognitive assessments, in the individualized group. Controlling for ATIII level at baseline and the initial score, the alteration in ATIII was positively correlated with changes in several scales, including HAMD-24 (r = 0.509, *p* = 0.016), SDS (r = 0.536, *p* = 0.010), SAS (r = 0.442, *p* = 0.039), and BHS (r = 0.479, *p* = 0.024), while a negative correlation was observed for change in Stroop C (r = −0.599, *p* = 0.003) (Figures 2A–E). Further multiple linear regression was utilized to verify these correlations, and supported that change in ATIII could affect changes in HAMD-24 and Stroop C after individualized rTMS (standardized β_HAMD−24_ = 0.423, *p* = 0.039; standardized β_StroopC_ = −0.471, *p* = 0.020).

**Figure 3:**
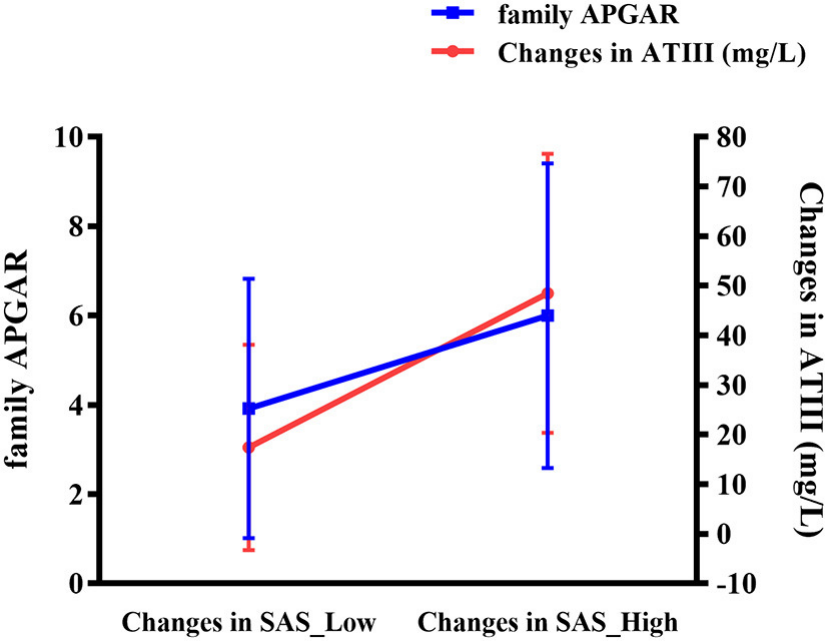
Interaction of altered ATIII (mg/L) and family APGAR on change in SAS in the individualized group. ATIII, antithrombin III; APGAR, Adaptation, Partnership, Growth, Affection, Resolve; SAS, Self-Rating Anxiety Scale.

Further analyses revealed an interactive effect of family APGAR and alteration in ATIII on change in SAS, indicating that family APGAR and change in ATIII affected change in SAS together in the individualized group (standardized β = 0.418, *p* = 0.042). As shown in Figure 3, patients with greater reduction in SAS showed more notable changes in ATIII and higher family APGAR scores.

### ROC Curve Analyses

Given the above-described findings, the ROC curve analysis used only ATIII rather than all four proteins. ATIII levels at two time points were analyzed for their ability to estimate or predict the response to 5 day treatment and remission at 4 week follow-up. In line with general practice, responders in this study were defined as patients with a change in HAMD-24 ≥50% and remission was evaluated by a HAMD-24 score ≤ 7.

**Figure 4:**
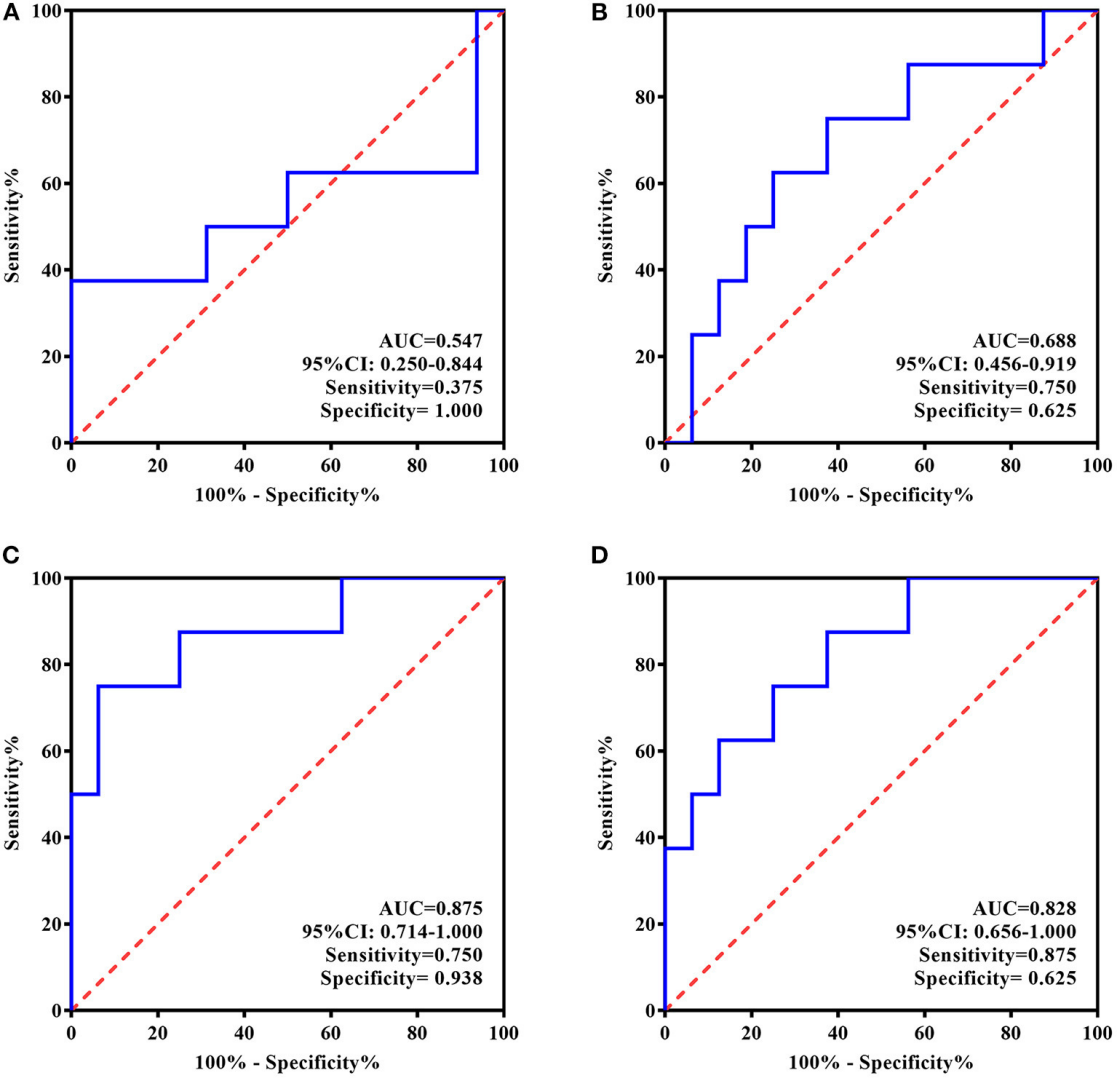
ROC Curve for the evaluating and predicting performance of ATIII in the individualized group. **(A)** ROC curve of ATIII at baseline for predicting the response to 5-day treatment; **(B)** ROC curve of ATIII at baseline for predicting the remission in 4 weeks following the treatment termination; **(C)** ROC curve of ATIII at day 5 for estimating the response to 5-day treatment; **(D)** ROC curve of ATIII at day 5 for predicting the remission in 4 weeks following the treatment termination. ROC curve, receiver operating characteristic curve; AUC, area under the curve; CI, confidence interval.

For the individualized group, larger areas under the curve (AUCs) were observed based on the ROC curves of ATIII after rTMS therapy. There was higher specificity for identifying responders to 5 day treatment (AUC = 0.875, 95% CI, 0.714–1.000, sensitivity = 0.750, specificity = 0.938) and higher sensitivity for predicting remission 4 weeks later (AUC = 0.828, 95% CI, 0.656–1.000, sensitivity = 0.875, specificity = 0.625). Nevertheless, ROC curve analyses of ATIII at baseline showed a low predictive performance for response and remission with small AUCs (AUC = 0.547, 95% CI, 0.250–0.844, sensitivity = 0.375, specificity = 1.000; AUC = 0.688, 95% CI, 0.456–0.919, sensitivity = 0.750, specificity = 0.625, respectively) (Figure 4).

**Figure 5:**
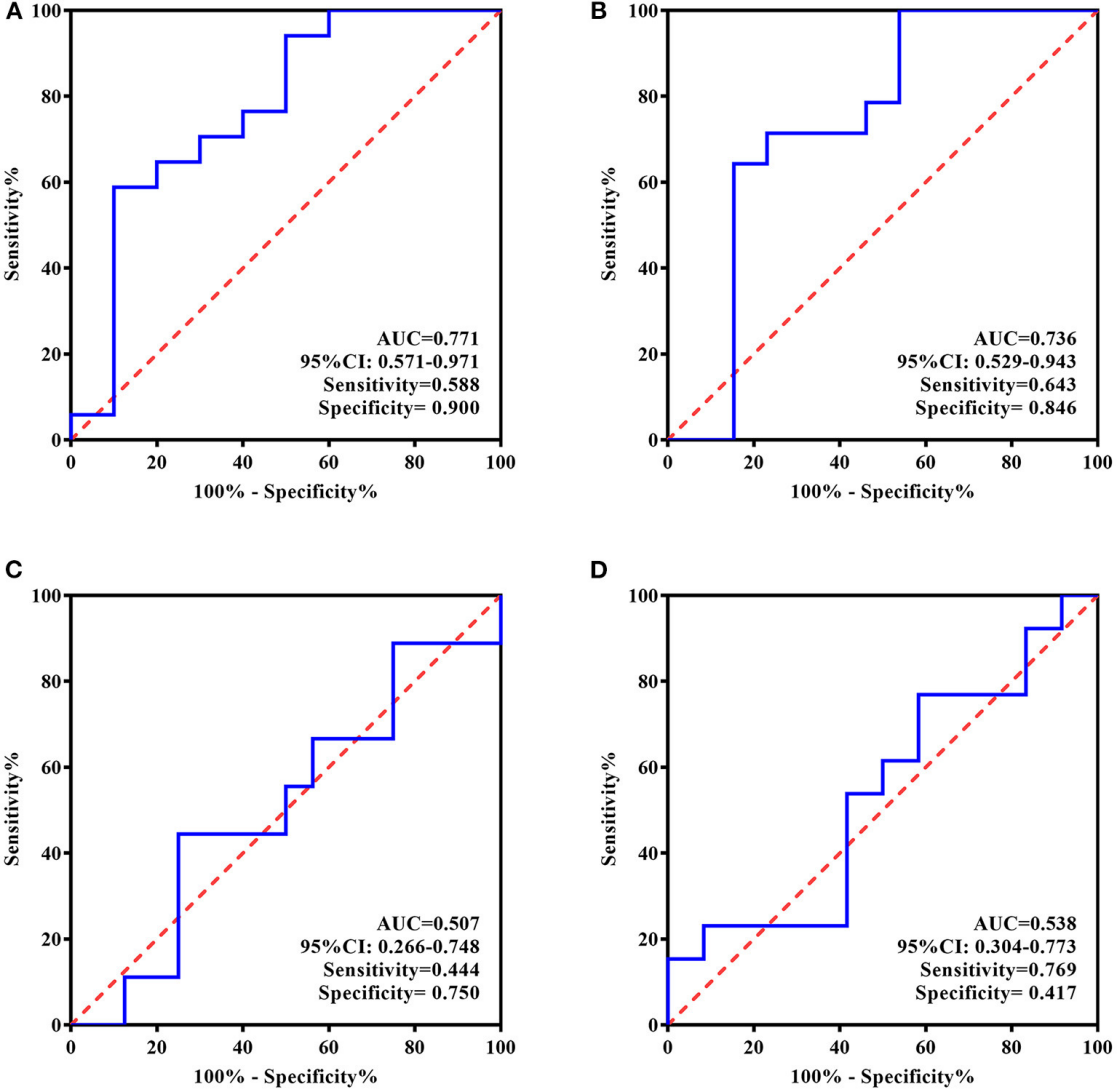
ROC Curve for the evaluating and predicting performance of ATIII in the standard group. **(A)** ROC curve of ATIII at baseline for predicting the response to 5-day treatment; **(B)** ROC curve of ATIII at baseline for predicting the remission in 4 weeks following the treatment termination; **(C)** ROC curve of ATIII at day 5 for estimating the response to 5-day treatment; **(D)** ROC curve of ATIII at day 5 for predicting the remission in 4 weeks following the treatment termination. ROC curve, receiver operating characteristic curve; AUC, area under the curve; CI, confidence interval.

In contrast, according to the ROC curves presented in Figure 5, baseline ATIII in the standard treatment group showed better efficiency for predicting rTMS antidepressive effects, represented by both response and long-term remission (AUC = 0.771, 95% CI, 0.571–0.971, sensitivity = 0.588, specificity = 0.900; AUC = 0.736, 95% CI, 0.529–0.943, sensitivity = 0.643, specificity = 0.846, respectively), whereas ATIII at the end of treatment was ineffective for the same predictions (AUC = 0.507, 95% CI, 0.266–0.748, sensitivity = 0.444, specificity = 0.750; AUC = 0.538, 95% CI, 0.304–0.773, sensitivity = 0.769, specificity = 0.417, respectively).

### Predictive Factors of Therapeutic Effects

Based on the assessments of affection at day 1, 3, and 5 (as well as additional assessments of HAMD-24 at week 1, 2, 3, 4 in the follow-up study) or cognitive tests after treatment, which were set as the curative effect indexes, multidimensional data were brought into the statistical model to estimate the potential predictive factors and their predictive values, including demographic and clinical information, days of treatment, treatment group, levels of the four proteins at baseline, and initial scores on all assessments and psychosocial scales. The value of baseline ATIII for predicting antidepressive efficacy is shown in Table 1. The antidepressive efficacy is supported by several assessments, such as HAMD-24 (*p* = 0.020), HAMA (*p* < 0.001), BSI-CV-C (*p* = 0.044), SDS (*p* < 0.001), SAS (*p* = 0.002), BHS (*p* = 0.014), Brief Assessment of Cognition in Schizophrenia: Symbol Coding (BACS SC, *p* = 0.002), and Stroop B (*p* = 0.048), while ITIH4 was only demonstrated to predict therapeutic effects represented by SAS (*p* = 0.044) (Supplementary Table 3). All potential predictive factors of each assessment are listed in Supplementary Table 3.

**Table 1:** ATIII at baseline involved in prediction of therapeutic effects.

	***t*** **score**	***p*** **-value**
HAMD-24	2.41	0.020*
HAMA	3.71	<0.001***
BSI-CV-C	2.07	0.044*
SDS	4.19	<0.001***
SAS	3.24	0.002**
BHS	2.57	0.014*
TMT-A	1.04	0.304
BACS SC	−3.29	0.002**
Stroop A	−1.61	0.114
Stroop B	−2.03	0.048*
NAB Mazes	−0.43	0.672
Stroop C	−1.41	0.167
BVMT-R	−1.01	0.316

* *p < 0.05*;

** * p < 0.01*;

*** *p < 0.001*.

*ATIII, antithrombin III; HAMD-24, 24-item Hamilton Depression Rating Scale; HAMA, Hamilton Anxiety Scale; BSI-CV-C, Beck Scale for Suicide Ideation-Chinese version-Current; SDS, Self-Rating Depression Scale; SAS, Self-Rating Anxiety Scale; BHS, Beck Hopelessness Scale; TMT-A, Trail Making Test A; BACS SC, Brief Assessment of Cognition in Schizophrenia: Symbol Coding; NAB Mazes, Neuropsychological Assessment Battery: Mazes; BVMT-R, Brief Visuospatial Memory Test-Revised*.

In addition, given the impacts of altered protein levels on long-term curative efficiency evaluated by HAMD-24 at week 1, 2, 3, and 4, additional data of changes in protein expression were included in the model. As shown in Table 2, both ATIII at baseline and change in ATIII were related to HAMD-24 assessments at follow-up (*p* = 0.024 and 0.023, respectively), supporting their power to predict later effect.

**Table 2:** Potential predictive factors of HAMD-24 in the follow-up study.

**Factor**	***t*** **score**	***p*** **-value**
ATIII at baseline	2.34	0.024*
Change in ATIII	2.35	0.023*
HAMD-24 at baseline	4.70	<0.001***
Days of treatment	−9.68	<0.001***
History of smoking	2.84	0.007**
CTQ-SF	2.24	0.030*

* *p < 0.05*;

** *p < 0.01*;

*** *p < 0.001*.

*ATIII, antithrombin III; HAMD-24, 24-item Hamilton Depression Rating Scale; CTQ-SF, Childhood Trauma Questionnaire-Short Form*.

## Discussion

The present study revealed downtrends in a panel of plasma proteins, namely CRP, ATIII, ITIH4, and VDB, after 5 day occipital rTMS treatment. Moreover, significant correlations between decrease in ATIII and improvements in neuropsychological assessments comprised of HAMD-24, SDS, SAS, BHS, and Stroop C were demonstrated in patients treated by individualized rTMS. This study also illustrated the predictive performance of ATIII, whether for rTMS treatment groups or all participants, suggesting the potential of this protein for estimating and predicting antidepressive efficacy.

There was no grouping bias in the affective assessments or levels of proteins, indicating that patients treated by different methods were in depressive states of similar clinical severity and had similar protein expression profiles at the group level at baseline. At the end of treatment, levels of three proteins, namely ATIII, ITIH4, and VDB, were decreased in both rTMS groups; a statistically significant reduction in CRP was only shown in the standard rTMS treatment group. As reported by Jha et al., reduced CRP was observed after 8 week sertraline treatment in MDD patients, in line with the findings of our study (54). However, alterations in the levels of the other three proteins after antidepressive treatment have not been investigated in previous studies. Of the four proteins, no notable differences were found in the sham group between baseline and day 5, suggesting the possible relevance of these proteins to the therapeutic process of occipital rTMS. Based on mounting evidence, inflammatory response is considered the common pathway of these four candidate biomarkers (55–58), and is speculated to be a promising target of multiple non-convulsive neurostimulation interventions, including rTMS (59, 60), which might partly account for the differences in protein alterations between rTMS and sham treatment.

As a member of the family of serine protease inhibitors (serpins), ATIII originates from *Serpin clade C, member 1 (SerpinC1)* with a reaction center for serine protease in the conservative spatial structure (61). Apart from its major suppressive roles in coagulation and hemostasis, ATIII exerts anti-inflammation action with or without mediation of anticoagulation (36, 56). A previous study detected higher ATIII mRNA levels in both gray and white matter in patients with Alzheimer's disease compared with controls (62); specifically, increased ATIII in astrocytes was speculated to be commensurate with astrogliosis, which has also been observed in MDD (63). Berk et al. reported a supersensitive platelet response to thrombin stimulation in depressed patients (64). A recent study of remitted MDD patients found a higher procoagulant index and fibrinogen level (65), suggesting an enhanced procoagulant state and a feasible mechanism for the increased risk of cardiovascular disease in MDD patients (66). ATIII can repress thrombin-induced activation of platelets and endotheliocytes and reduce their secretions, such as interleukin-1 and P-selectin, which are demonstrated to promote interactions between endothelial cells and neutrophils to further aggravate inflammation (67, 68). As reported by Stelzhammer et al., compared to baseline levels, ATIII in MDD patients increased significantly after first ECT treatment; however, this was identified by liquid-chromatography mass spectrometry and not in combination with multiplex immunoassay (37). Our inconsistent results might be ascribed to several reasons. Firstly, only 10 patients with treatment-resistant depression were analyzed in the study by Stelzhammer et al. without expanded and independent validation. Such a low sample size might result in a false positive, as the authors speculated, and differences in race and antidepressant resistance could exacerbate inconsistencies. Moreover, increased ATIII was observed in patients treated by ECT after just once session, indicating its involvement in acute ECT effect. By contrast, in the present study, reduced ATIII levels were detected after 5 day rTMS treatment. The diverse therapeutic courses and related pathways could lead to the different protein expression profiles.

As determined in the present study, the decline in ATIII was further associated with improvement of clinical symptoms comprising depression, anxiety, hopelessness, and cognitive deficiency in the individualized group. Moreover, for the same patients, an interaction with change in SAS was demonstrated between altered ATIII and family APGAR, suggesting that family support may impact the relationship between protein levels and curative effect. On a deeper level, biological and environmental factors could interact to affect the antidepressive efficiency of occipital rTMS.

A biomarker should be useful for identifying and/or predicting response to treatment. In the present study, baseline ATIII in the standard group and decreased ATIII after individualized rTMS showed a high performance for estimating or predicting antidepressive effect, suggesting the prognostic value of ATIII at both baseline and the end of rTMS treatment. As a key factor, baseline ATIII was involved in the prediction of all emotional assessments, cognitive tests of information processing speed, and long-term efficacy evaluated by HAMD-24 at follow-up study together with changes in ATIII. Taken together, these findings demonstrate that ATIII could be a potential biomarker for curative effects in MDD treatment, regardless of the therapeutic method.

Previous proteomic research identified four proteins connected to MDD (28). In the present study, following rigorous scientific research, one of the four candidates, ATIII, was identified as a potential biomarker of both MDD diagnosis and antidepressive effect evaluation and forecasting by multi-verification. As ATIII level can be detected by a simple test, it is suitable for clinical screening. Another indicator identified in the proteomic study, VDB, was validated both *in vitro* and *in vivo* and an application has been filed for a patent. For this reason, we believe it is worthwhile to verify ATIII in a larger cohort and with further animal and cell experiments, followed by developing a convenient and efficient kit and filing a patent.

There are several limitations of this work. Firstly, this study explored biomarkers of curative efficacy based on the rapid antidepressive effects of occipital rTMS by analyzing changes in ATIII before and after rTMS treatment. As most MDD patients took medication or completed other therapies as prescribed at follow-up, homogeneity could not be maintained. In the light of this, blood samples were only collected at baseline and after 5 day rTMS stimulation. Due to the lack of blood collection at 4 week follow-up, it was not possible to assess changes in ATIII during this period or to evaluate its ability to estimate the long-term efficacy of antidepressive treatment. However, we were still able to demonstrate the predictive value of ATIII using a statistical model. Secondly, considering the importance of patient safety and need for patient cooperation, MDD patients who were suicidal or in a stuporous state were excluded. Therefore, our findings are limited to patients with moderate to severe depression without extreme negative symptoms. Thirdly, in order to eliminate interference from antidepressants and increase homogeneity, this study required patients to be drug naïve or drug free for at least 2 weeks before recruitment and reconfirmed MDD diagnosis at follow-up before statistics. In a future study, we will add rTMS stimulation along with standard medication to expand the scope of significance of ATIII. We will also carry out a non-inferiority study to compare the antidepressive effects of occipital and prefrontal rTMS in which there is no restriction related to the use of antidepressant drugs. Lastly, the small sample size could increase the risk of false-positive results; to validate the findings of this study, it is necessary to repeat this analysis in an independent cohort with a larger sample.

## Conclusion

In conclusion, this study revealed decreases in CRP, ATIII, ITIH4, and VDB after occipital rTMS therapy. Furthermore, we revealed a relationship between greater reductions in ATIII and greater improvements in neuropsychological assessments in patients who received individualized stimulation. Ultimately, we demonstrated the potential value of ATIII as a biomarker of MDD and antidepressive treatment outcomes.

## Data Availability Statement

The raw data analyzed in this article are not publicly available. Requests to access the data should be directed to janemengzhang@vip.163.com.

## Ethics Statement

The studies involving human participants were reviewed and approved by the Ethics Committee of the Second Affiliated Hospital of Xinxiang Medical University. The patients/participants provided their written informed consent to participate in this study.

## Author Contributions

ZZ conceived and designed the study. ZZ and HoZ supervised to carry out the protocols and enrolled subjects. ZZ, HoZ, and RS characterized subjects. RS collected specimens, analyzed data, prepared the tables and figures, wrote the manuscript, and followed ZZ's guidance and they were responsible for the data interpretation. HaZ, KL, and BW were responsible for multi-mode MRI scan. XL, JZ, and YY carried out rTMS treatment. YS measured levels of CRP, ATIII, ITIH4, and VDBP. YZ established the linear mixed model. LG and YS assisted with the structure design of the manuscript. All authors contributed to the article and approved the submitted version.

## Funding

The work was funded by the National Key Research and Development Plan of China (Nos. 2016YFC1306700 and 2016YFC1306704), the National Natural Science Key Foundation of China (No. 81830040), Science and Technology Program of Guangdong (No. 2018B030334001), Program of Excellent Talents in Medical Science of Jiangsu Province (No. JCRCA2016006), the Program for One Thousand Zhongyuan Talents (No. 204200510020), and NSFC-Henan Mutual Funds (U1704190).

## Conflict of Interest

The authors declare that the research was conducted in the absence of any commercial or financial relationships that could be construed as a potential conflict of interest.

## Publisher's Note

All claims expressed in this article are solely those of the authors and do not necessarily represent those of their affiliated organizations, or those of the publisher, the editors and the reviewers. Any product that may be evaluated in this article, or claim that may be made by its manufacturer, is not guaranteed or endorsed by the publisher.
