# Supplementary material for: Potential of Antithrombin III as a Biomarker of Antidepressive Effect in Major Depressive Disorder
Source: Front Psychiatry. 2021 Oct 28;12:678384. doi: 10.3389/fpsyt.2021.678384 (PMC8580946; doi:10.3389/fpsyt.2021.678384)
Supplement: Supplementary file 1 [file Data_Sheet_1.docx]

**Supplementary file**

**Supplementary Figure 1**


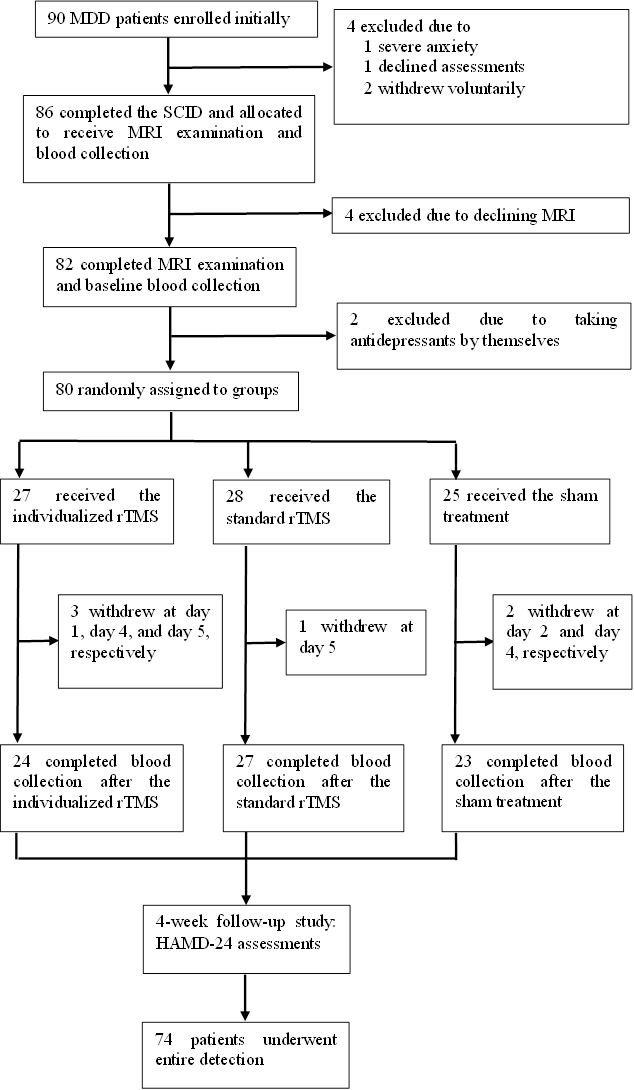


**Supplementary Figure 1.** Flowchart of study design. MDD = major depressive disorder; SCID = the Structured Clinical Interview for the Diagnostic and Statistical Manual of Mental Disorders, Fourth Edition (DSM-IV); MRI = magnetic resonance imaging; rTMS = repetitive transcranial magnetic stimulation; HAMD-24 = 24-item Hamilton Depression Rating Scale.

| **Supplementary Table 1. Demographic and clinical characteristics of MDD patients in different treatment groups.** | | | | | |
| --- | --- | --- | --- | --- | --- |
|  | **Individualized**  **Group**  **(n=24)** | **Standard**  **Group (n=27)** | **Sham**  **Group**  **(n=23)** | **Score** | **p Value** |
| **Age, years,**  **mean (SD)** | 31.71 (12.92) | 31.33 (12.77) | 31.61 (10.49) | 0.007 | 0.993^a^ |
| **Gender (M/F), n** | 11/13 | 10/17 | 10/13 | 0.438 | 0.803^b^ |
| **Education years,**  **mean (SD)** | 12.92 (2.77) | 11.67 (3.88) | 12.43 (3.80) | 0.816 | 0.446^a^ |
| **BMI,**  **mean (SD)** | 22.82 (3.64) | 22.95 (3.60) | 21.94 (3.30) | 0.591 | 0.557^a^ |
| **History of drinking, n** | 0 | 3 | 4 | 6.239 | 0.044^b,*^ |
| **History of smoking, n** | 7 | 4 | 2 | 3.569 | 0.168^b^ |
| **Family history of mental disease, n** | 5 | 12 | 9 | 3.342 | 0.188^b^ |
| **Age of onset, years,**  **mean (SD)** | 29.50 (13.31) | 27.74 (12.13) | 25.13 (7.55) | 0.532 | 0.766^c^ |
| **First episode/ Relapse, n** | 19/5 | 17/10 | 16/7 | 1.605 | 0.448^b^ |
| **Frequency of onset, median (IQR)** | 1 (1-1.25) | 1 (1-2) | 1 (1-2) | 0.922 | 0.631^c^ |
| **Total course, months, median (IQR)** | 16 (5-33) | 24 (12-72) | 24 (3-132) | 1.512 | 0.469^c^ |
| **Current course, months, median (IQR)** | 5 (2-14) | 6 (1-12) | 6 (2-36) | 0.853 | 0.653^c^ |
| **Drug naïve/ Drug free, n** | 14/10 | 13/14 | 16/7 | 2.341 | 0.310^b^ |
| Data are presented as mean (SD) or median (IQR) or absolute numbers.  ^a^ One-way ANOVA.  ^b^ Chi-square test.  ^c^ Kruskal-Wallis test.  *p<0.05.  Abbreviations: MDD = major depressive disorder, M = male, F = female, BMI = body mass index, SD = standard deviation, IQR = interquartile range. | | | | | |

| **Supplementary Table 2. Neuropsychological assessments in different groups at baseline and the end of treatment.** | | | | | | | | | | |
| --- | --- | --- | --- | --- | --- | --- | --- | --- | --- | --- |
|  | **Individualized Group (n=24)** | | | **Standard Group (n=27)** | | | **Sham Group (n=23)** | | | **p Value** |
|  | **Baseline** | **Day 5** | **p Value**  **Baseline vs. Day 5** | **Baseline** | **Day 5** | **p Value Baseline vs. Day 5** | **Baseline** | **Day 5** | **p Value Baseline vs. Day 5** | **comparison among groups at baseline** |
| **Emotional assessments** | | | | | | | | | | |
| **HAMD-24,**  **mean (SD)** | 33.79 (6.31) | 14.79 (9.47) | <0.001^a,***^ | 35.81 (7.91) | 20.15 (10.47) | <0.001^a,***^ | 35.70 (9.28) | 23.22 (10.01) | <0.001^a,***^ | 0.606^c^ |
| **HAMA,**  **mean (SD)** | 25.92 (7.59) | 11.42 (7.87) | <0.001^a,***^ | 28.15 (9.06) | 14.89 (9.59) | <0.001 ^a,***^ | 26.83 (9.98) | 16.17 (9.90) | <0.001^a,***^ | 0.669^c^ |
| **BSI-CV-C,**  **median (IQR)** | 1 (0-7.25) | 0 (0-0.75) | <0.001^b,***^ | 3  (1-16) | 0  (0-1) | <0.001^b,***^ | 1  (0-5) | 0  (0-2) | 0.005^b,**^ | 0.111^d^ |
| **SDS,**  **mean (SD)** | 66.83 (10.63) | 53.21 (16.33) | <0.001^a,***^ | 70.56 (12.00) | 55.07 (18.83) | <0.001 ^a,***^ | 73.35 (11.05) | 58.09 (14.40) | <0.001^a,***^ | 0.146^c^ |
| **SAS,**  **mean (SD)** | 61.33 (9.74) | 48.29 (13.36) | <0.001^a,***^ | 62.63 (14.89) | 49.70 (16.23) | <0.001 ^a,***^ | 63.61 (13.50) | 51.74 (12.30) | <0.001^a,***^ | 0.834^c^ |
| **BHS,**  **mean (SD)** | 11.04 (4.30) | 6.83 (5.24) | <0.001^a,***^ | 10.33 (4.76) | 7.22 (6.24) | <0.001 ^a,***^ | 11.52 (5.55) | 9.00 (5.86) | 0.003^a,**^ | 0.687^c^ |
| **Psychosocial assessments** | | | | | | | | | | |
| **LES,**  **mean (SD)** | 51.54 (41.22) | NA | NA | 38.81 (34.70) | NA | NA | 48.87 (33.33) | NA | NA | 0.424^c^ |
| **CTQ-SF,**  **mean (SD)** | 54.71 (11.25) | NA | NA | 46.52 (8.92) | NA | NA | 54.74 (12.42) | NA | NA | 0.010^c,*^ |
| **family APGAR,**  **mean (SD)** | 4.96 (3.28) | NA | NA | 6.33 (3.04) | NA | NA | 4.26 (2.85) | NA | NA | 0.056^c^ |
| **SCSR,**  **mean (SD)** | 42.13 (8.53) | NA | NA | 45.41 (9.94) | NA | NA | 43.74 (13.66) | NA | NA | 0.561^c^ |
| **Cognitive assessments** | | | | | | | | | | |
| **Information processing speed** | | | | | | | | | | |
| **TMT-A,**  **mean (SD)** | 47.29 (11.86) | 34.75 (9.18) | <0.001^a,***^ | 41.75 (13.96) | 32.43 (9.65) | <0.001^a,***^ | 39.87 (14.83) | 28.91 (11.21) | <0.001^a,***^ | 0.156^c^ |
| **BACS SC,**  **mean (SD)** | 46.21 (10.97) | 49.50 (11.68) | 0.007^a,**^ | 48.23 (10.83) | 51.81 (12.67) | 0.021^a,*^ | 49.95 (11.91) | 53.86 (8.50) | 0.019^a,*^ | 0.529^c^ |
| **Stroop A,**  **mean (SD)** | 80.29 (21.20) | 86.29 (24.85) | 0.023^a,*^ | 87.38 (19.93) | 94.15 (18.84) | 0.015^a,*^ | 95.64 (20.37) | 100.77 (23.40) | 0.085^a^ | 0.046^c,*^ |
| **Stroop B,**  **mean (SD)** | 63.33 (16.25) | 69.96 (18.19) | 0.003^a,**^ | 70.31 (21.11) | 76.04 (18.85) | 0.005^a,**^ | 75.48 (16.66) | 81.96 (19.74) | 0.010^a,*^ | 0.079^c^ |
| **Executive function** | | | | | | | | | | |
| **NAB Mazes,**  **mean (SD)** | 9.63 (6.59) | 13.67 (8.66) | <0.001^a,***^ | 11.08 (8.30) | 12.54 (8.13) | 0.108^a^ | 9.59 (6.91) | 11.95 (8.43) | 0.006^a,**^ | 0.719^c^ |
| **Stroop C,**  **mean (SD)** | 36.21 (11.71) | 44.21 (13.51) | <0.001^a,***^ | 38.88 (12.21) | 45.81 (13.74) | <0.001^a,***^ | 45.73 (16.78) | 54.59 (12.95) | 0.003^a,**^ | 0.058^c^ |
| **Visuospatial memory and learning function** | | | | | | | | | | |
| **BVMT-R,**  **mean (SD)** | 23.00 (7.61) | 25.63 (6.01) | 0.014^a,*^ | 24.58 (8.52) | 25.15 (8.50) | 0.599^a^ | 23.48 (6.94) | 25.09 (7.76) | 0.084^a^ | 0.762^c^ |
| Data are presented as mean (SD) or median (IQR).  ^a^ paired t-test.  ^b^ Wilcoxon signed-rank test.  ^c^ One-way ANOVA.  ^d^ Kruskal-Wallis test.  *p<0.05, **p<0.01, ***p<0.001.  Abbreviations: HAMD-24 = 24-item Hamilton Depression Rating Scale, HAMA = Hamilton Anxiety Scale, BSI-CV-C = Beck Scale for Suicide Ideation-Chinese version-Current, SDS = Self-Rating Depression Scale, SAS = Self-Rating Anxiety Scale, BHS = Beck Hopelessness Scale, LES = Life Event Scale, CTQ-SF = Childhood Trauma Questionnaire-Short Form, APGAR = Adaptation, Partnership, Growth, Affection, Resolve, SCSR = Self-Consciousness Scale Revised, TMT-A = Trail Making Test A, BACS SC = Brief Assessment of Cognition in Schizophrenia: Symbol Coding, NAB Mazes = Neuropsychological Assessment Battery: Mazes, BVMT-R = Brief Visuospatial Memory Test-Revised, NA = not applicable, SD = standard deviation, IQR = interquartile range. | | | | | | | | | | |

| **Supplementary Table 3. Potential predictive factors of affective and cognitive assessments.** | | | |  |
| --- | --- | --- | --- | --- |
|  | **Factor** | **t/F Score** | **p Value** |  |
| **HAMD-24** | | | |  |
|  | **Gender** | -2.27 | 0.028^*^ |  |
|  | **History of smoking** | 2.53 | 0.015^*^ |  |
|  | **Days of treatment** | -17.12 | <0.001^***^ |  |
|  | **Grouping** | 4.82 | 0.013^*^ |  |
|  | **ATIII at baseline** | 2.41 | 0.020^*^ |  |
|  | **HAMD-24 at baseline** | 5.70 | <0.001^***^ |  |
| **HAMA** | | | |  |
|  | **Days of treatment** | -10.44 | <0.001^***^ |  |
|  | **ATIII at baseline** | 3.71 | <0.001^***^ |  |
|  | **HAMA at baseline** | 7.84 | <0.001^***^ |  |
| **BSI-CV-C** | | | |  |
|  | **BMI** | 2.26 | 0.029^*^ |  |
|  | **History of drinking** | -2.90 | 0.006^**^ |  |
|  | **Days of treatment** | -2.31 | 0.026^*^ |  |
|  | **Grouping** | 3.73 | 0.032^*^ |  |
|  | **ATIII at baseline** | 2.07 | 0.044^*^ |  |
|  | **BSI-CV-C at baseline** | 9.43 | <0.001^***^ |  |
|  | **SCSR** | -3.02 | 0.004^**^ |  |
| **SDS** | | | |  |
|  | **History of drinking** | 2.02 | 0.049^*^ |  |
|  | **Days of treatment** | -5.21 | <0.001^***^ |  |
|  | **ATIII at baseline** | 4.19 | <0.001^***^ |  |
|  | **SDS at baseline** | 4.72 | <0.001^***^ |  |
| **SAS** | | | |  |
|  | **Days of treatment** | -4.47 | <0.001^***^ |  |
|  | **ATIII at baseline** | 3.24 | 0.002^**^ |  |
|  | **ITIH4 at baseline** | -2.07 | 0.044^*^ |  |
|  | **SAS at baseline** | 5.04 | <0.001^***^ |  |
| **BHS** | | | |  |
|  | **History of drinking** | 2.52 | 0.015^*^ |  |
|  | **Days of treatment** | -5.05 | <0.001^***^ |  |
|  | **ATIII at baseline** | 2.57 | 0.014^*^ |  |
|  | **BHS at baseline** | 8.30 | <0.001^***^ |  |
| **TMT-A** | | | |  |
|  | **Age, years** | 2.37 | 0.022^*^ |  |
|  | **Gender** | -2.52 | 0.016^*^ |  |
|  | **History of smoking** | 2.64 | 0.011^*^ |  |
|  | **TMT-A at baseline** | 3.13 | 0.003^**^ |  |
| **BACS SC** | | | |  |
|  | **ATIII at baseline** | -3.29 | 0.002^**^ |  |
|  | **BACS SC at baseline** | 6.48 | <0.001^***^ |  |
|  | **LES** | 2.54 | 0.015^*^ |  |
| **Stroop A** |  |  |  |  |
|  | **Stroop A at baseline** | 5.90 | <0.001^***^ |  |
| **Stroop B** | | | |  |
|  | **Age, years** | -2.24 | 0.030^*^ |  |
|  | **Total course, months** | 2.47 | 0.018^*^ |  |
|  | **ATIII at baseline** | -2.03 | 0.048^*^ |  |
|  | **Stroop B at baseline** | 8.73 | <0.001^***^ |  |
| **NAB Mazes** | | | |  |
|  | **NAB Mazes at baseline** | 10.74 | <0.001^***^ |  |
| **Stroop C** | | | |  |
|  | **Age, years** | -2.27 | 0.028^*^ |  |
|  | **Age of onset, years** | 2.05 | 0.046^*^ |  |
|  | **Total course, months** | 2.70 | 0.010^**^ |  |
|  | **Frequency of onset** | -2.49 | 0.017^*^ |  |
|  | **Stroop C at baseline** | 5.33 | <0.001^***^ |  |
| **BVMT-R** | | | |  |
|  | **Education years** | 2.82 | 0.007^**^ |  |
|  | **BVMT-R at baseline** | 5.22 | <0.001^***^ |  |
| *p<0.05;  **p<0.01;  ***p<0.001.  Abbreviations: BMI = body mass index, ATIII = antithrombin III, ITIH4 = inter-alpha-trypsin inhibitor heavy chain H4, HAMD-24 = 24-item Hamilton Depression Rating Scale, HAMA = Hamilton Anxiety Scale, BSI-CV-C = Beck Scale for Suicide Ideation-Chinese version-Current, SDS = Self-Rating Depression Scale, SAS = Self-Rating Anxiety Scale, BHS = Beck Hopelessness Scale, TMT-A = Trail Making Test A, BACS SC = Brief Assessment of Cognition in Schizophrenia: Symbol Coding, NAB Mazes = Neuropsychological Assessment Battery: Mazes, BVMT-R = Brief Visuospatial Memory Test-Revised, LES = Life Event Scale, SCSR = Self-Consciousness Scale Revised. | | | |  |
